# Optical surface guidance for submillimeter monitoring of patient position during frameless stereotactic radiotherapy

**DOI:** 10.1002/acm2.12611

**Published:** 2019-05-16

**Authors:** Elizabeth L. Covington, John B. Fiveash, Xingen Wu, Ivan Brezovich, Christopher D Willey, Kristen Riley, Richard A. Popple

**Affiliations:** ^1^ Department of Radiation Oncology University of Alabama‐Birmingham Birmingham AL USA; ^2^ Department of Neurosurgery University of Alabama‐Birmingham Birmingham AL USA

**Keywords:** surface guided radiotherapy, stereotactic radiosurgery, intrafraction motion, optical surface imaging, frameless radiosurgery

## Abstract

**Purpose:**

To evaluate the accuracy of monitoring intrafraction motion during stereotactic radiotherapy with the optical surface monitoring system. Prior studies showing a false increase in the magnitude of translational offsets at non‐coplanar couch positions prompted the vendor to implement software changes. This study evaluated two software improvements intended to address false offsets.

**Methods:**

The vendor implemented two software improvements: a volumetric (ACO) rather than planar calibration and, approximately 6 months later, an improved calibration workflow (CIB) designed to better compensate for thermal drift. Offsets relative to the reference position, obtained at table angle 0 following image‐guided setup, were recorded before beam‐on at each table position and at the end of treatment the table returned to 0° for patients receiving SRT.

**Results:**

Prior to ACO, between ACO and CIB, and after CIB, 223, 155, and 436 fractions were observed respectively. The median magnitude of translational offsets at the end of treatment was similar for all three intervals: 0.29, 0.33, and 0.27 mm. Prior to ACO, the offset magnitude for non‐zero table positions had a median of 0.79 mm and was found to increase with increasing distance from isocenter to the anterior patient surface. After ACO, the median magnitude was 0.74 mm, but the dependence on surface‐to‐isocenter distance was eliminated. After CIB, the median magnitude for non‐zero table positions was reduced to 0.57 mm.

**Conclusion:**

Ongoing improvements in software and calibration procedures have decreased reporting of false offsets at non‐zero table angles. However, the median magnitude for non‐zero table angles is larger than that observed at the end of treatment, indicating that accuracy remains better when the table is not rotated.

## 1 INTRODUCTION

Frameless radiosurgery has been used to improve patient comfort during treatment and has been shown to have the same level of positional accuracy as framed treatments.^1^, ^2^, ^3^, ^4^ Our institution has previously published on the use of FFF‐VMAT stereotactic radiotherapy (SRT) to significantly reduce treatment time 5 as well as utilizing a single isocenter for the treatment of multiple brain metastases.^6^ Due to the use of a headrest and mask in place of an invasive head‐frame, imaging during treatment via x‐ray or optical guidance has been used to monitor intrafraction motion.^4^, ^7^, ^8^ We recently commissioned an Edge (Varian Medical Systems, Palo Alto, CA) linear accelerator with an optical surface monitoring system (OSMS), a camera based system for surface guided radiation therapy (SGRT), to monitor all SRT patients for intrafraction motion.

Optical surface guidance has been used for patient positioning and monitoring for a range of clinical sites,^9^, ^10^, ^11^, ^12^ including intracranial SRS.^4^, ^13^ Detailed descriptions of this technology have been previously published^14^ and only a basic overview is presented here. OSMS uses three pods, each with two cameras and a projector, to monitor the patient during treatment. The projector is used to project a pseudo‐speckle pattern on the patient that is used for the three‐dimensional reconstruction of the patient’s position. The patient is monitored with a reference surface, called the region‐of‐interest (ROI), which is created from the patient’s CT scan or can be captured before treatment begins. During treatment, the OSMS system presents the deviations from this reference surface, which are referred to as real time deltas (RTDs). OSMS displays the lateral, longitudinal, and vertical offsets as well as rotation offsets in pitch, roll, and yaw. Treatment thresholds can be set that will gate the beam if these thresholds are exceeded.

In this paper, we present the aggregate data of intrafraction motion during frameless FFF‐VMAT SRT captured via OSMS before and after two updates provided by the vendor: (a) a vendor performed volumetric, rather than planar, calibration, called advanced camera optimization (ACO) and (b) a user performed cold camera state calibration detailed in a customer information bulletin (CIB). The procedure recommended by the CIB was designed to account for thermal drift. Our goals in this study were to assess the OSMS reported patient movement for a large cohort of SRT patients as well as monitor any improvement in perceived falsely reported offsets at non‐zero table angles after the aforementioned software updates. RTDs were used to compare aggregate data of intrafraction patient motion before and after implementation of ACO and CIB.

## 2 MATERIALS AND METHODS

Previous reports have shown that OSMS experiences a drift in RTDs during monitoring of stationary phantoms.^15^ Li et al reported that the vertical delta drifted ~0.4 mm in the first 10 min of monitoring of a stationary object while lateral and longitudinal deltas were ~0.1 mm. We hypothesized that this behavior was due to temperature changes in the camera pods caused by the speckle pattern projectors. To investigate this behavior, we tracked the RTDs of a stationary object and the temperatures reported by the camera pods for 30 min after turning on the projectors.

**Figure 1:**
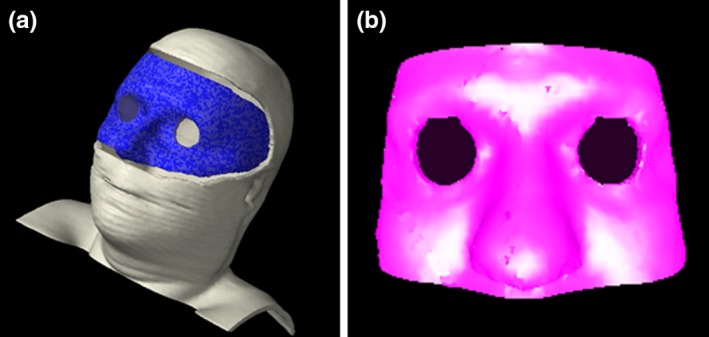
(left) Reference surface (in blue) created in the treatment planning system by contouring the open portion of the mask and subtracting a 0.5 cm margin and removing the eye region. (right) The imported reference surface in optical surface monitoring system is saved as a region of interest.

Intrafraction tracking with OSMS requires the use of an open facemask to allow creation of reference surface for motion tracking. Due to reports of inaccuracy related to insufficient or problematic ROIs,^8^ we developed a workflow for the creation of ROIs within the treatment planning system. This helps prevent the inclusion of the thermoplastic mask within the ROI and led to a standardized practice for ROI creation. During simulation, the outer edge of the open face region of the thermoplastic mask is lined with radiopaque wire. These wires are used to visualize the border of the mask in the contouring workspace and ensure that the ROI excludes the mask. In the contouring workspace, a structure is created from the entire portion of the face that is enclosed by the wires. This contour is then modified by removing a 0.5 cm border from the perimeter of the structure to exclude any mask material. The eye region is also removed to reduce any fluctuations in RTDs from eye movement. A representative contour of an ROI is shown in Fig. 1 in both the contouring workspace and the OSMS software. This ROI structure is then exported to the OSMS software.

**Figure 2:**
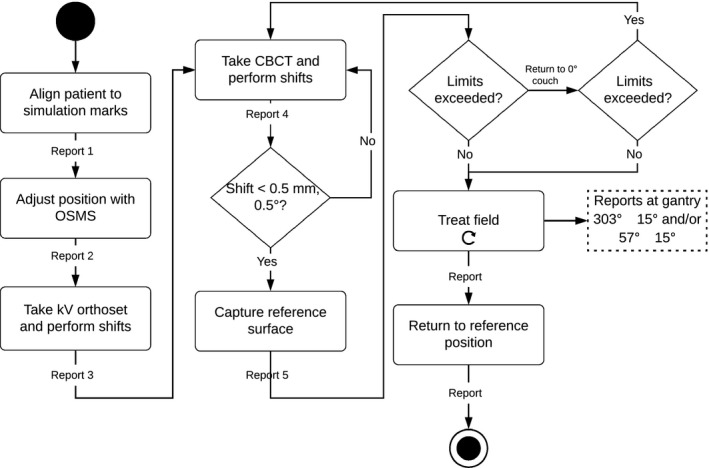
Treatment workflow and report capture time points for radiosurgery. When motion limits are exceeded, the patient is moved back to the reference position. If the action limits are still exceeded, another CBCT is taken. Patients are returned to the reference position at the end of treatment to record the final positional offset

Version 5.0.1747 of the OSMS software did not support the continuous recording of RTDs. In order to record the RTDs throughout treatment, OSMS reports were used to save RTDs at designated time points. When the “Report” button is pushed, a screenshot of the OSMS software is captured along with the RTDs and saved into a Microsoft Word document. The following workflow, shown in Fig. 2, was created to enable a systematic data collection method of RTDs during treatment. Report 1 is captured after the patient is aligned on the table with alignment marks from simulation. The patient’s position is then corrected using the OSMS RTDs. The mask can be removed to adjust for rotational offsets to minimize deviations from the DICOM ROI before imaging. Once the therapists are satisfied that they have minimized the RTDs, report 2 is captured. An orthogonal kV imaging set is taken, shifts are applied and report 3 is captured. A CBCT is performed, and shifts are applied. All shifts are done with 6° of freedom (6DOF). If shifts in any direction are greater than 0.5 mm and/or 0.5° are indicated, these are applied and another scan is taken. If the shifts are below this threshold, the shifts are applied and report 4 is taken. An updated reference surface is now captured with OSMS and all RTDs should be approximately zero in report 5. For each treatment field, a report is taken before beam on, during gantry motion when a camera pod is blocked (at approximately 57°±15° and/or 303°±15°), and when the beam is completed. After report 4, reports are numbered consecutively and their field number and time point (before beam‐on, gantry angle, etc) are documented by the attending physicist on a patient‐specific form. If the RTD thresholds are exceeded, the beam is turned off and treatment is resumed if the RTDs return to sub‐threshold values. If not, the couch is returned to zero and the RTDs are monitored. If RTDs at couch zero are within tolerance, the couch is returned to the treatment angle and the field is delivered. If RTDs are approximately zero at couch zero but are exceeded at the couch angle, then the threshold is updated to reflect the offset of the baseline position at the specific couch angle. For example, if the magnitude (MAG) of RTDs is zero at couch zero but 0.7 mm at couch 45°, the threshold for beam off would be 1.7 mm for a 1 mm threshold. If the RTDs remain above the thresholds at couch zero, a CBCT is performed, shifts applied, and a new reference is captured. Automatic beam gating it not utilized; rather the RTDs are monitored by the clinical team where the beam is manually turned off if RTDs exceed the patient‐specific thresholds. Thresholds were typically 1 mm, but may have been reduced due to proximity of organs at risk or for functional SRS patients (e.g., trigeminal neuralgia, thalamotomy). At the end of treatment, the couch is returned to the reference position at couch zero and a final report is taken. To assess the accuracy of reported RTDs, RTDs were analyzed at the following time points: (a) before beam on for each field, (b) during gantry motion when a camera pod was blocked, and (c) at the end of treatment at the original reference position.

All later software versions had continuous logging of RTDs and the report capture button was removed. For each monitoring session, the RTDs were recorded in a text file saved in a patient‐specific directory at the end of the session. In lieu of manual report capture to record RTDs at points of interest during treatment (e.g., before beam on), the log files were correlated with treatment records obtained from the oncology information system (OIS).

Numerous phantom studies have been performed to assess the accuracy of OSMS.^1^, ^15^, ^16^, ^17^, ^18^, ^19^, ^20^ To characterize the accuracy of our system prior to ACO and CIB, we wanted to determine which portion of the RTDs seen at non‐zero couch angles were due to couch walkout. Couch walkout was evaluated by both dial gauge and MV imaging (EPID). In addition, an anthropomorphic polystyrene head (Floracraft, Ludington, MI) phantom with a tungsten BB, placed either directly upon the ROI or posterior surface, was used to assess the magnitude of couch walkout in comparison to the RTDs reported by OSMS. A region of interest was chosen to best represent a typical radiosurgery ROI. The BB was placed at isocenter with an eight‐gantry angle MLC Winston‐Lutz test with the phantom positioned by a 3‐axis micrometer translation stage. Every 22.5°, an MV image and OSMS report was taken. The offset of the BB from isocenter was monitored via EPID as the couch was rotated. This offset was considered couch walkout. These data were used to see if the RTDs correlated with couch walkout and if the depth of isocenter affected the accuracy of RTDs at non‐zero couch angles.

Our OSMS system was upgraded with a feature that was under a limited evaluation release during the time period of this study. It is now currently being deployed to all systems with software version 5.1 and current systems are being updated. Advanced Camera Optimization (ACO) is performed by the company rather than the user performed isocenter calibration. Rather than calibrating to a single plane with the calibration plate, the camera optics are calibrated over a volume to improve the accuracy of the surface tracking with a precision manufactured ACO calibration plate. We repeated the phantom studies post ACO to determine if there was improvement in tracking at non‐zero couch angles. We also repeated the phantom studies with a mid‐plane BB after CIB to determine if any improvement in accuracy was seen in RTDs at non‐zero couch angles. Aggregate patient data were also compared prior and post ACO and CIB to look for improvement in the increased RTDs observed at non‐zero couch angles.

## 3 RESULTS

**Figure 3:**
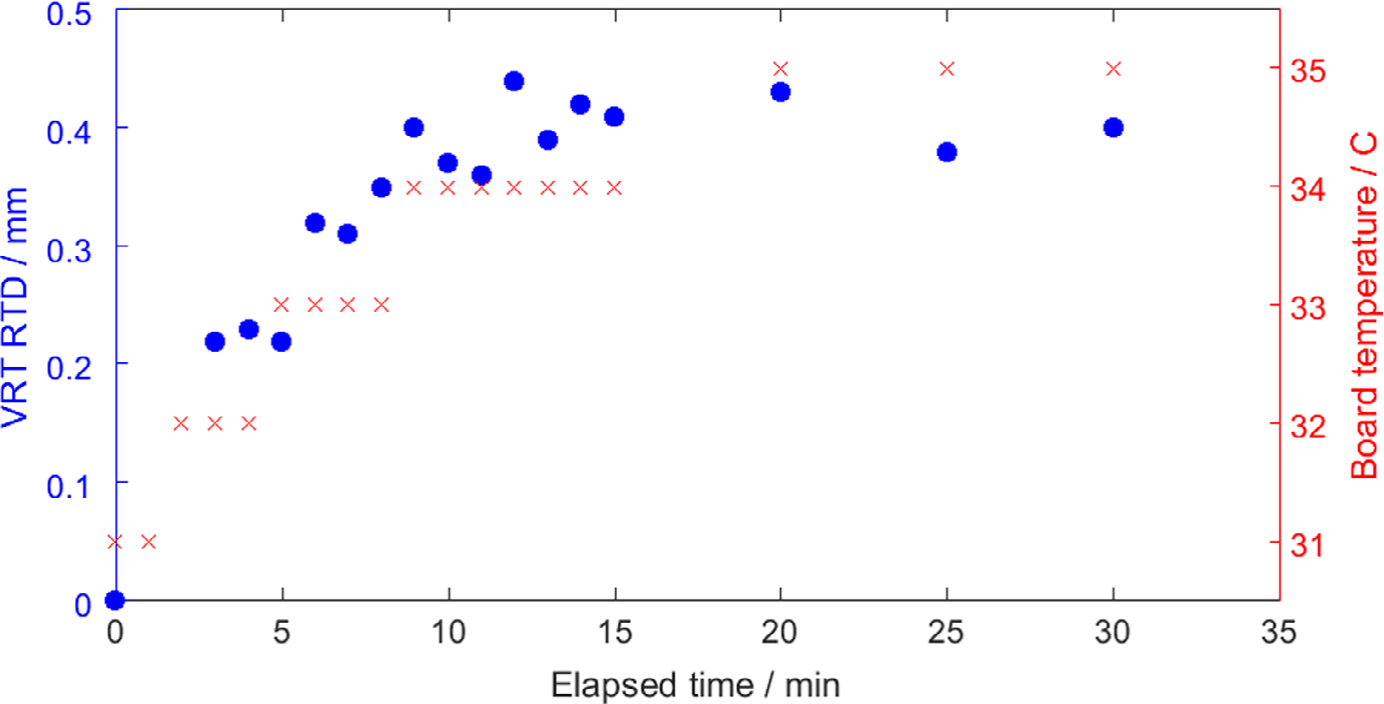
Drift of vertical real time delta over time for a stationary object (blue) and the change in the camera pod board temperature (red). The magnitude of the drift is mostly comprised of changes in the vertical direction and stabilizes after approximately 15 min.

For a stationary phantom monitored for 30 min, we found the average magnitude (MAG), the root mean square of translational RTDs, of RTDs to be 0.5 mm that was composed primarily of an offset in the vertical direction. This behavior also was found to be correlated with the reported board temperature on the camera pods. Figure 3 shows the change in the vertical RTD and board temperature as a function of time. The drift plateaus around 15 min and remains stable thereafter. Note that the drift begins only when the projector is on and the speckle pattern is being displayed and is not related to how long the camera pods have been powered on.

Prior to ACO, between ACO and CIB, and after CIB, 223, 155, and 436 fractions delivered by flattening filter free (FFF) beams on an Edge linear accelerator using OSMS were evaluated, respectively. The 223 fractions reported in the initial patient study (prior to ACO) were taken from a larger dataset of 271 reports captured, where 38 were omitted for either incomplete data or irresolvable inconsistencies (e.g., number of reports did not match number of treatment fields, OSMS software crashed mid‐treatment, multiple reference captures). Of the 30 omitted, five were due to multiple reference surface captures from CBCT confirmed patient motion. Between ACO and CIB, we omitted 21 fractions of out 176 for similar reasons including 11 treatments with mid‐treatment imaging and a new reference surface capture due to patient movement (6.5%). Post CIB, 66 of 502 fractions were omitted, with 24 (4.8%) having multiple reference captures. The focus of this study was to monitor for improvement of falsely reported increase of RTDs at non‐zero couch angles; therefore, patients that had repeat imaging were excluded due to having multiple reference surfaces that precluded the comparison of patient position throughout treatment. Out of 941 fractions monitored in this study, 40 (4.3%) had CBCT confirmed movement that required a new reference capture.

**Figure 4:**
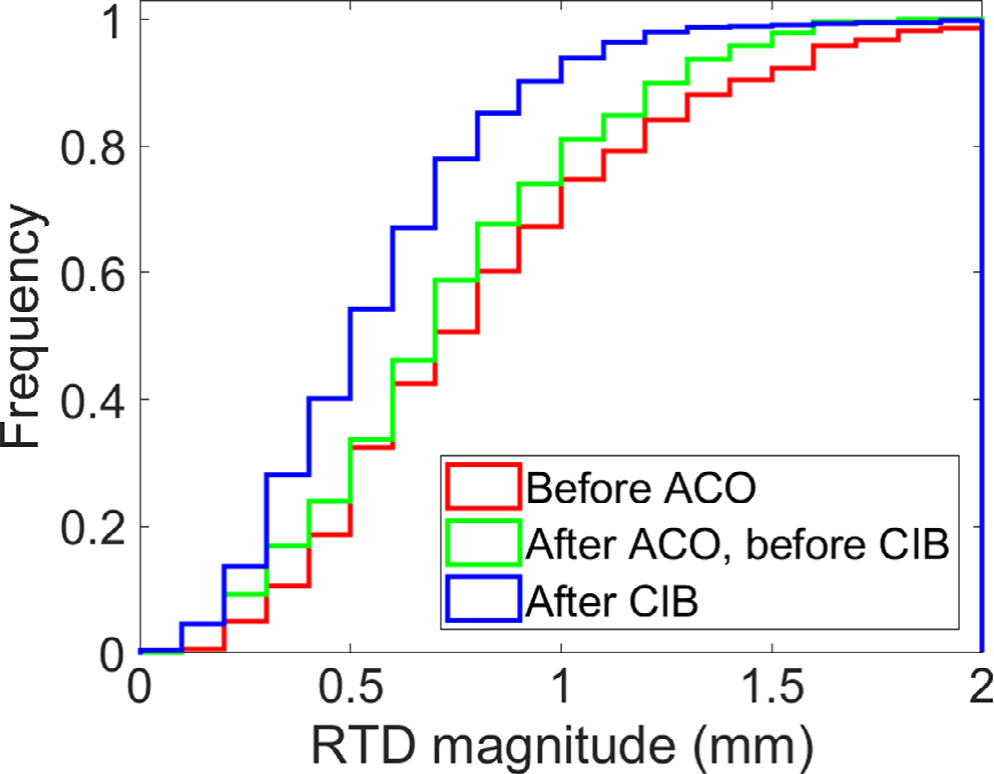
Cumulative histogram of the magnitude of real time deltas (RTDs) for the three time periods: (red) before advanced camera optimization (ACO), (green) after ACO but before customer information bulletin (CIB), and (blue) after CIB before beam‐on at non‐zero table angles

The mean time between reference capture and end of treatment was 4.84 min (range 1.15–37.27 min). The median magnitude at the end of treatment was similar for all three intervals: 0.29, 0.33, and 0.27 mm. The interquartile range (IQR) for the three intervals, respectively, were 0.29, 0.31, and 0.25 mm. Prior to ACO, the translational magnitude for non‐zero table positions before beam‐on had a median of 0.79 mm (IQR 0.55) and was found to increase with increasing distance from isocenter to the anterior patient surface. After ACO, the median magnitude was 0.74 mm (IQR 0.52), but the dependence on isocenter location was eliminated. After CIB, the median magnitude for non‐zero table positions was reduced to 0.57 mm (IQR 0.39). The difference before and after the CIB between the median magnitude of the RTD at non‐zero table positions was statistically significant, with a Wilcoxon rank sum test *P* = 0.00. Figure 4 shows the cumulative histogram of the magnitude of translational RTDs for the three periods before beam on at non‐zero table angles. RTDs during gantry motion, when the gantry head blocked the camera pod, were not found to be different from RTDs recorded before beam‐on with all cameras unobscured. The mean magnitude was 0.85 mm before beam‐on and during gantry rotation when a camera pod was blocked.

To investigate if the increase in RTDs at non‐zero couch angles were falsely reported, the translational magnitude of RTDs were compared before beam‐on at non‐zero couch angles and at the end of treatment at couch zero. We focused on RTDs exceeding 1 mm at non‐zero couch angles to exclude submillimeter magnitudes due to couch walkout. A treatment fraction was deemed to have a false positive for patient motion if the RTD magnitude exceeded 1 mm at non‐zero couch angles but the end of treatment couch zero RTD magnitude was <0.5 mm. The incidence of false positives was shown to improve after ACO and CIB. Prior to ACO, the false positive rate was 32.8% (57 of 174, 95% confidence interval 25.8% to 40.3%). After ACO and prior to CIB, the false positive rate was 29.7% (33 of 111, 95% confidence interval 21.4% to 39.1%). After CIB, the false positive rate was reduced to 11.5% (41 of 357, 95% confidence interval 8.4% to 15.3%).

**Figure 5:**
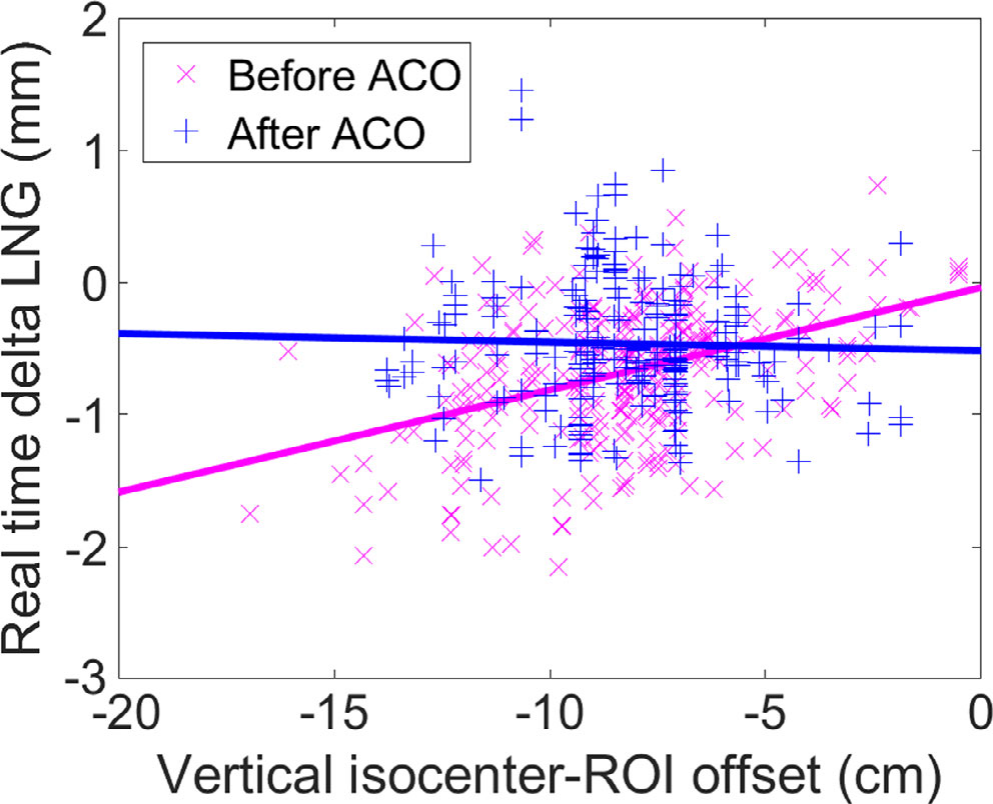
Longitudinal real time delta increases as the distance between the centroid of the region‐of‐interest (ROI) and isocenter increases at non‐zero couch angles prior to advanced camera optimization (ACO). This dependence is eliminated after ACO. (Before ACO: slope = 0.077 mm/cm, intercept = −0.04 mm (*R* ^2^ = 0.173); After ACO: slope = −0.006 mm/cm, intercept = −0.52 mm, *R* ^2^ = 0.001).

The largest component of the translation offset was found to be in the longitudinal direction. Couch walkout was investigated as a potential source of the increased longitudinal offset, but the magnitude of longitudinal walkout was <0.4 mm when evaluated via dial gauge.^21^ Variability in longitudinal RTDs amongst patients also suggested a cause other than couch walkout. The distance between the ROI and isocenter was investigated as a potential cause of interpatient variation. Figure 5 shows the longitudinal RTD versus vertical distance between the centroid of the ROI and the isocenter prior to ACO (×) and after ACO (+). As this distance increases, the magnitude of the offset also increases prior to ACO. After ACO, this behavior was no longer present. Anecdotally reported fluctuations in RTDs during gantry motion, caused by camera pod blockage by the gantry head, were not found to be statistically significant from RTDs recorded before beam‐on.

**Figure 6:**
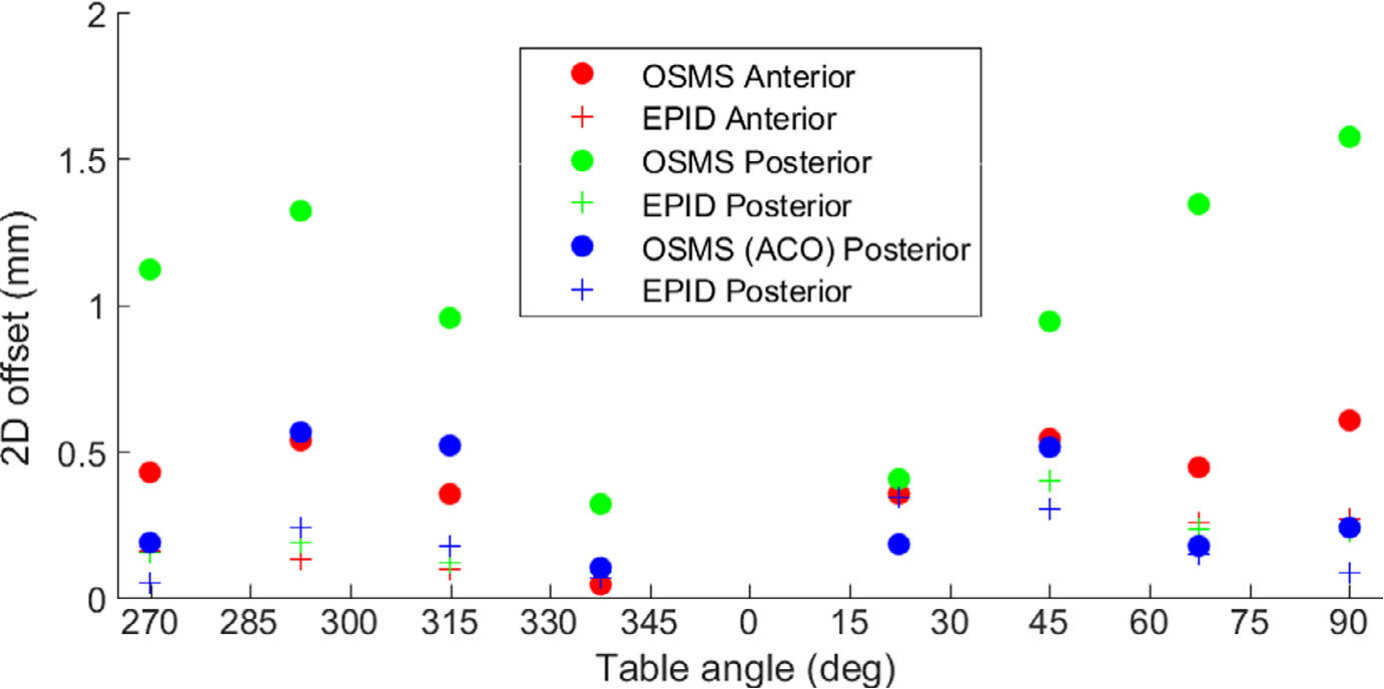
A comparison of the two‐dimensional (2D) offset of a tungsten BB in an anthropomorphic phantom determined by either optical surface monitoring system (OSMS) (circles) or EPID (crosses). Anterior indicates the BB has zero vertical offset between the ROI surface and isocenter while the posterior BB has a 19 cm offset. After advanced camera optimization (ACO), the 2D offset for a posterior target decreases to that of an anterior target.

To further investigate this trend, studies were done with an anthropomorphic phantom having various distances between the ROI surface and isocenter. The EPID measured walkout was compared to the OSMS reported RTDs of the ROI as shown in Fig. 6. Anterior refers to a BB located on the surface of the phantom where there is zero vertical offset between the isocenter and ROI while posterior refers to a BB embedded on the back on the phantom with a 19 cm offset between isocenter and the ROI. The couch walkout observed via EPID is small with maximum values under 0.5 mm while the OSMS reported offsets approached 1.7 cm when the ROI was 19 cm above isocenter. Similar to the patient data, the OSMS reported offsets increase with couch angle and increasing vertical distance between the ROI and isocenter. The studies where the ROI was 19 cm above isocenter were repeated after ACO. Figure 6 shows an improvement in the OSMS reported two‐dimensional offset which are now comparable to when the ROI was at the plane of isocenter.

**Figure 7:**
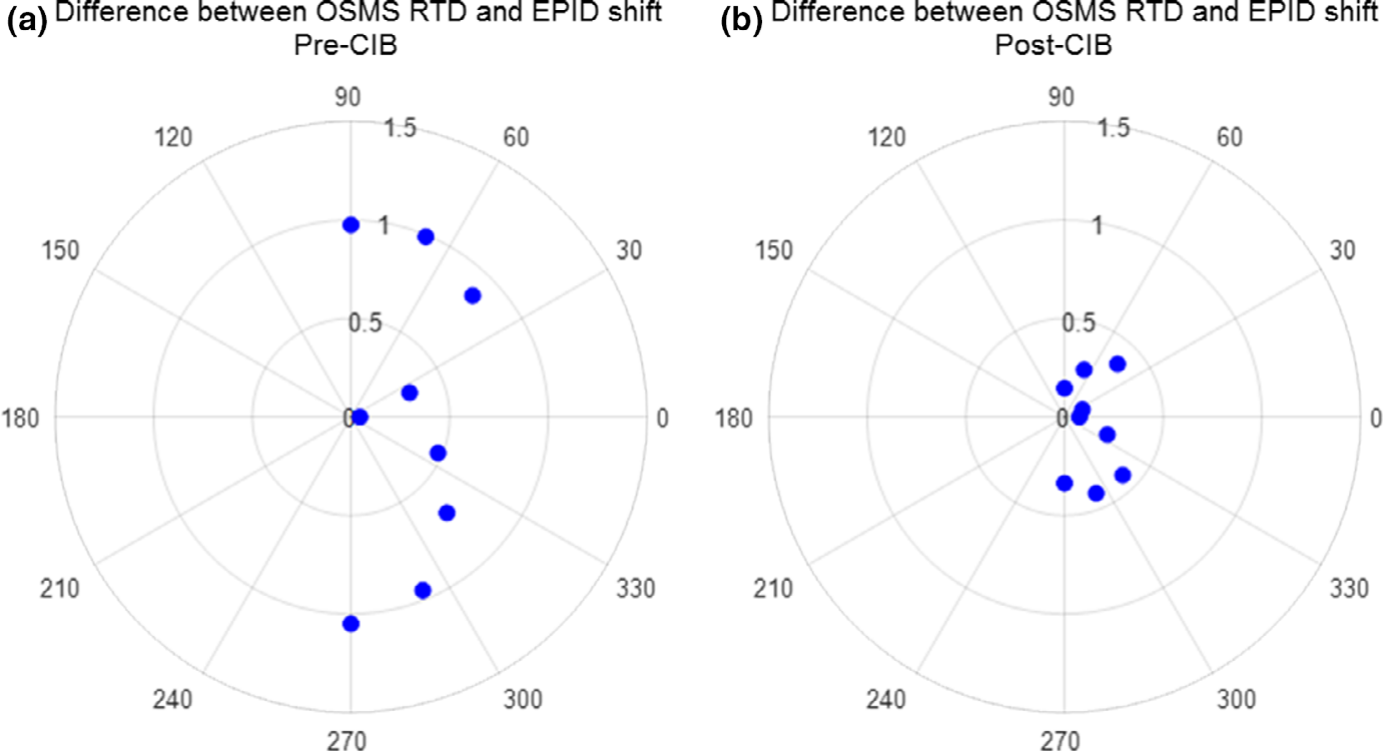
Difference between real time deltas (RTDs) and EPID measured shifts at various couch angles for an anthropomorphic phantom with bb at isocenter located 8 cm below region‐of‐interest (ROI). Customer information bulletin (CIB) calibration reduced the optical surface monitoring system (OSMS) reported shifts where (a) prior to CIB they exceed 1 mm to (b) <0.5 mm.

After the release of the CIB, we repeated the phantom study with a bb at midplane depth approximately 8 cm below the ROI surface. Figure 7(a) shows the difference in the OSMS reported RTDs and the EPID reported shift before and after CIB. Before CIB, the differences can exceed 1 mm at various couch angles. Recalibrating with the CIB guidelines reduced the differences to under 0.5 mm, as shown in Fig. 7(b), for all couch angles.

## 4 DISCUSSION

The OSMS reported offsets show a thermal drift that begins when projectors are turned on during monitoring. To prevent this offset from being perceived as patient motion during treatment, therapists have been advised to turn on the projector (i.e., start monitoring) before the patient enters the room and monitor for a minimum of 10 min before using the system for alignment. Therapists are also advised to compare the ambient versus board temperature on pods. The drift has been shown to stabilize when the board temperature is approximately 5° higher than the ambient temperature. To prevent this behavior from affecting system performance, projectors are left in a “cold” state for calibration, which is detailed in the CIB. Rather than turning on the projectors, single images are captured to prevent the thermal drift of RTDs. As seen in the phantom studies, this led to a reduction in the RTDs at non‐zero couch angle resulting in submillimeter differences in RTDs and EPID reported shifts. Although cameras are calibrated in the cold state, we recommend treating with a warm camera pods to avoid having the thermal drift perceived as patient motion during treatment.

The magnitude of patient motion from beginning to end of treatment reported by OSMS in the present study is consistent with previous reports of patient motion observed using CBCT and other kV‐based image guidance. Seravalli et al. performed post treatment CBCTs for 59 treatments and found an average translation magnitude of 0.6 mm.^22^ Badakhshi et al. found an average translational intrafraction deviation of 1 mm using kV imaging with BrianLAB for 190 patients over 269 treatments.^7^ While both measured offsets larger than those reported in this study, the average timescale was reported to be 15 min in both studies versus 4.84 min in this study. Overall, OSMS was capable of recording submillimeter deviations from the patient’s initial reference surface from beginning to end of treatment.

While the magnitude of motion recorded by OSMS at the end of treatment was consistent with previous studies, larger than expected RTDs were observed during treatment, particularly at non‐zero couch angles. This behavior was the impetus for phantom studies on the perceived inaccuracy of OSMS at non‐zero couch angles. Our phantom studies confirmed that the RTDs reported at non‐zero couch angles are not consistent with the location determined by on‐board imaging prior to ACO. The deviation from isocenter for the tungsten BB observed by portal imaging was of submillimeter magnitude and attributed to couch walkout. Corresponding OSMS reported deviations often exceed 1.5 mm and increased as the location of isocenter increased in distance from the reference surface. These results indicate that patients with posterior lesions may require additional observation during treatment to determine if OSMS reported deltas are truly indicative of patient movement in OSMS systems that have not undergone the ACO upgrade. It should also be noted that the direction of OSMS reported deltas did not always correlate with imaging studies. For example, a posterior BB was found to have a 0.2 mm offset longitudinally via portal imaging while OSMS reported a −0.8 mm longitudinal delta. After ACO, the magnitude and direction of RTDs and couch walkout were still not found to be correlated.

Due to these inconsistencies, it is not advised to adjust patient position based on OSMS reported RTDs at any couch angle. Our practice is to confirm any exceeded tolerances observed at non‐zero couch angles by returning the patient to couch zero and use CBCT to adjust the patient position. Even for posterior targets, the deviations between OSMS and imaging studies at couch zero were in submillimeters; therefore, the couch zero RTDs can be used to differentiate anomalies seen at non‐zero couch angles from actual patient movement. If RTDs continue to exceed thresholds at couch zero, CBCT imaging can be repeated and a new reference surface captured. OSMS is never used to adjust the position of the patient. It should be noted that all of our patients are aligned with 6DOF couch alignment; therefore, both translational and rotational offsets have been accounted for during radiographic image guidance and capturing of the OSMS reference position. These data in this manuscript focus on the magnitude of translational offsets reported in OSMS that does not include rotational offsets in distance from isocenter calculation. For clinics without 6DOF alignment, patients’ alignment with only translational adjustment may have different results from the data presented here due to residual error in the MAG values due to unresolved rotational misalignment.

We believe that the behavior exhibited by OSMS is intrinsic to the camera pod geometry that increases inaccuracies in the longitudinal, and to a lesser degree lateral, direction as the monitored object is rotated around the isocenter. It is possible that there could be performance differences between systems due to variations in camera pod configuration and arrangement. Each system would need to be independently investigated and compared. To ensure that the camera positions have not changed since calibration, OSMS requires daily QA be done before treatment where deviations must be within 1 mm. For monthly QA, our system was recalibrated if the isocenter calibration showed deviations above 0.3 mm. Due to all treatment being delivered after passing daily QA along with monthly checks of the isocenter calibration, we do not believe that slight changes in camera position are responsible for the behaviors seen in this study. It should be noted that our patient data are biased to particular couch angles. Of the 1358 RTDs captured for non‐coplanar beams, the most common table angle was 45° (22.8%), followed by 315° (21.6%), 70° (19.1%), 90° (12.5%), and 290° (8.5%). In addition, 62.2% of the plans were on the 90° couch side and 37.8% were on the 270° side. Clinics that use other couch angles may experience different outcomes.

Our recommendations for utilizing surface guidance imaging with OSMS for intrafraction patient motion during SRT are detailed in Table 1. These recommendations have emerged after several years of experience and treating over 1000 fractions of SRT with OSMS. Our future work will be a continuation of our infraction data collection to assess the performance of the system and continual evaluation of software and system upgrades. Based on our experience to date, we strongly recommend utilizing OSMS only as a screening tool for patient motion and positioning patients exclusively with radiographic image guidance.

**Table 1:** Recommendations for intrafraction motion monitoring during stereotactic radiotherapy with optical surface monitoring system

Issue	Recommendations
Thermal drift	Determine time for thermal drift equilibrium Turn on projectors before patient treatment
Couch walk‐out	Measure couch walk‐out (EPID, dial gauge, etc.) Phantom study to compare walk‐out with real time deltas (RTDs)
RTD dependence on surface‐to‐isocenter distance	Advanced camera optimization upgrade
Increase in RTDs at non‐zero couch angles	Perform cold calibration detailed in customer information bulletin
RTD exceeds thresholds at non‐zero couch angles	Return couch to zero and evaluate RTDs
RTD exceeds thresholds at couch zero	Repeat radiographic image guidance

## 5 CONCLUSION

Ongoing improvements in software and calibration procedures have decreased reporting of false offsets at non‐zero table angles. However, the median magnitude for non‐zero table angles is larger than that observed at the end of treatment, indicating that accuracy remains better when the table is not rotated. While OSMS can be used to monitor patients for gross motion during SGRT, it is not recommended to reposition patients with OSMS reported offsets during intracranial SRT.

## CONFLICTS OF INTEREST

No conflicts of interest.
